# The Relationship between Internet Patient Satisfaction Ratings and COVID-19 Outcomes

**DOI:** 10.3390/healthcare11101411

**Published:** 2023-05-12

**Authors:** Jonathan Stanley, Mark Hensley, Ronald King, Neil Baum

**Affiliations:** 1 Vanguard Communications, Denver, CO 80205, USA; 2 Hensley Biostats, Seattle, WA 98102, USA; 3 Tulane Medical School, Tulane University, New Orleans, LA 70112, USA

**Keywords:** COVID-19, patient-reported outcomes, patient satisfaction ratings, online reviews, healthcare outcomes

## Abstract

Our prior research showed that patient experience—as reported by Google, Yelp, and the Hospital Consumer Assessment of Healthcare Providers and Systems survey—is associated with health outcomes. Upon learning that COVID-19 mortality rates differed among U.S. geographic areas, we sought to determine if COVID-19 outcomes were associated with patient experience. We reviewed daily, U.S.-county-level-accrued COVID-19 infections and deaths during the first year of the pandemic using each locality’s mean online patient review rating, correcting for county-level demographic factors. We found doctor star ratings were significantly associated with COVID-19 outcomes. We estimated the absolute risk reduction (ARR) and relative risk reduction (RRR) for each outcome by comparing the real-world-observed outcomes, observed with the mean star rating, to the outcomes predicted by our model with a 0.3 unit higher average star rating. Geographic areas with higher patient satisfaction online review ratings in our models had substantially better COVID-19 outcomes. Our models predict that, had medical practices nationwide maintained a 4-star average online review rating—a 0.3-star increase above the current national average—the U.S may have experienced a nearly 11% lower COVID-19 infection rate and a nearly 17% lower death rate among those infected.

## 1. Introduction

Patient experience ratings have received increased attention in healthcare, but their significance is still being assessed. Since 2006, patient-reported experiences after hospitalization have been collected using the Hospital Consumer Assessment of Healthcare Providers and Systems (HCAHPS) survey. The HCAHPS survey is the current nationwide standard for patient-experience-of-care data [1].

Additionally, Patient Online Reviews (PORs) are a vast and potentially rich source of information for large-scale analysis [2]. POR websites, such as Yelp and Google, enable patients to rate their healthcare providers with a star rating between one and five stars, with one star being the worst and five stars being the best. These internet testimonials are elaborate [3], free, continuously updated, and often reveal the specific causes of a patient’s experience [4]. PORs not only mirror many aspects of the HCAHPS survey but also reflect new areas of importance to patients and caregivers that may have significant implications for policy makers [5]. A study of hospitals found 90% of patient review narratives commented on clinicians and staff, which were overwhelmingly positive, and 52% commented on hospital facilities, such as hospital cleanliness, food, parking, and amenities [6]. A study of nursing homes found the most common theme in online reviews was regarding staff caring (53%) [7].

In addition to measuring patient experience, PORs have shown substantial associations to health outcomes and can be used as a data source for understanding healthcare quality [8]. For example, Yelp ratings were associated with lower readmission rates for all conditions and lower mortality for myocardial infarction and pneumonia [9]. Web-based positive recommendations of hospitals were shown to be significantly associated with lower hospital standardized mortality ratios [10] and they contained key themes for emergency care [11]. PORs have been associated with the resolution of original complaints [12] and geographically to key measures of healthcare coordination and quality [2]. PORs can also be used to enhance the evidence base for general decision making in healthcare [13].

In 2014, we developed the Happy Patient Index (HPI), which assessed Google and Yelp PORs by locality [14]. We used automated computer software to catalog all available Google and Yelp PORs for businesses explicitly identified as doctors with an address within 50 miles of the city center, as defined by Google Maps, for each of the 100 most populous cities within the U.S. The resulting HPI dataset contained over 46,000 PORs, which were used to determine the average POR star rating for each of the localities, herein to be referred to as the Locality Mean Patient Online Rating (LMPOR). These were found to be as low as 3.20 stars and as high as 4.15 stars on a scale of 1–5. Wealth—or a lack thereof—did not appear to affect LMPOR in the HPI; three of the top-10 happiest areas had mean household incomes below the national mean.

Upon learning that COVID-19 mortality rates differed among U.S. geographic areas, we sought to determine if COVID-19 outcomes were associated with LMPORs.

## 2. Materials and Methods

### 2.1. Data Sources

We obtained daily U.S.-county-level-accrued COVID-19 infections and deaths from the Centers for Disease Control and Prevention (CDC) and state- and local-level public health agencies as compiled by USA Facts for the first year of the COVID-19 pandemic (between 11 March 2020 and 11 March 2021) [15].

We obtained LMPORs for 100 U.S. localities from the HPI dataset. These were the most recent source of LMPORs available at the time.

We obtained the selected characteristics of county-level data from the 2015–2019 American Community Survey (ACS) 5-Year Estimates from the U.S. Census Bureau, including population, demographics [16], selected economic characteristics [17] and selected social characteristics [18]. These were the most recent estimates available at the time. Rates were reported as a proportion of total county population to which the descriptor applied.

**Figure 1:**
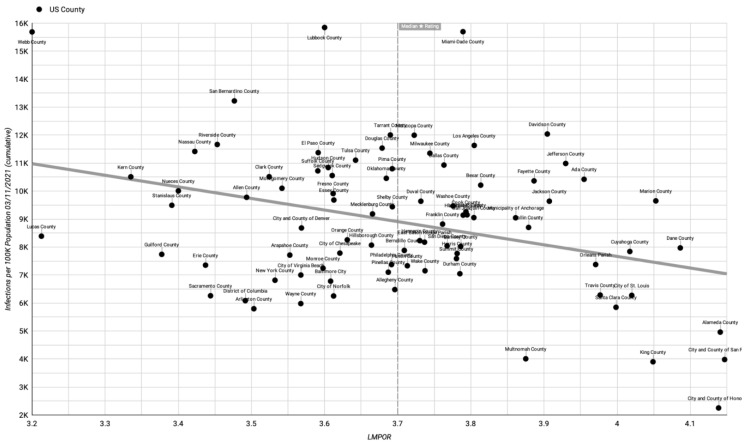
Uncorrected LMPOR vs. cumulative COVID-19 infections per 100k population until 11 March 2021.

**Figure 2:**
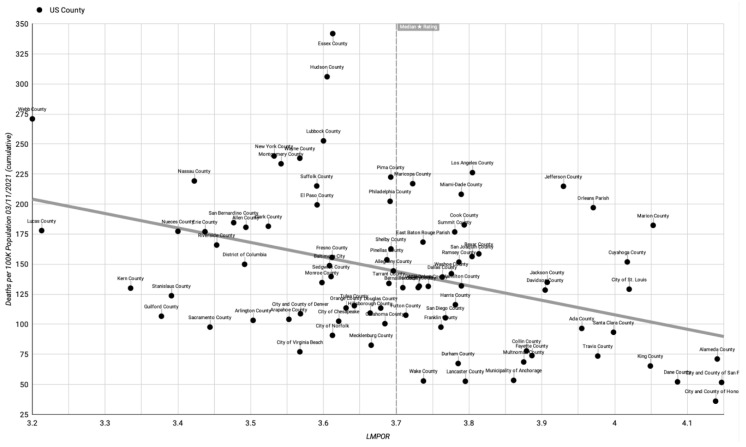
Uncorrected LMPOR vs. cumulative COVID-19 deaths per 100k population until 11 March 2021.

**Figure 3:**
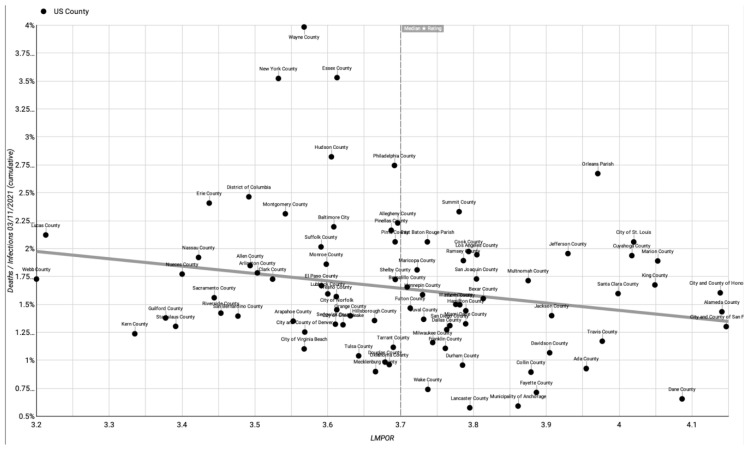
Uncorrected LMPOR vs. cumulative COVID-19 deaths/infections until 11 March 2021.

The raw data (Table S1) can be used to produce a scatter plot of outcomes with a linear trendline. For example, Figure 1, Figure 2 and Figure 3, respectively, show LMPOR versus the deaths per 100k population, LMPOR versus infections per 100k population, and LMPOR versus the infected death rate for the counties (all as of 31 March 2021 and prior to correction for possible confounders).

### 2.2. Statistical Analysis

We matched the LMPOR to its respective county-level information. Since LMPORs contained PORs from a 50-mile radius, some LMPORs had a substantially overlapping area and were effectively clones. For example, we had overlapping LMPORs for Phoenix, Scottsdale, Mesa, Chandler, and Glendale areas, all being in Maricopa County, Arizona. In those instances, we avoided over-representing those duplicative LMPORs by only matching the county-level outcomes to its most populous/recognizable locality. For example, we matched Maricopa County COVID-19 outcomes only to the Phoenix area LMPOR (which represents all PORs within 50 miles of the Phoenix epicenter and is inclusive of the other cities mentioned). This had the effect of removing from our dataset entries for which the predictor variables (star ratings) are exactly the same, and would otherwise have exerted undue influence on our overall results. This process eliminated 11 of the original 100 cities, resulting in a total of 89 localities for our analysis dataset.

We determined the daily COVID-19 outcome rates from county-level data: accrued deaths divided by accrued infections (the infected death rate), accrued deaths divided by population (the population death rate), and accrued infections divided by population (the population infection rate).

We used this complete dataset to measure the outcome for the first year of the pandemic. Furthermore, we investigated the results from the first three months of the pandemic as well as the remainder of its first year in consideration of the novelty of the disease during the first wave and the subsequently evolving public health response thereafter:

1. First year of the pandemic: 11 March 2020–11 March 2021;
2. Initial pandemic: 11 March 2020–11 June 2020;
3. Later pandemic: 11 June 2020–11 March 2021.

Additionally, we investigated several smaller time periods reflecting post hoc knowledge of the wave-like changes in disease incidence over time. Respectively, these periods represent a five month relative lull, a two month rising wave, and a two month falling wave:

1. Summer/Fall pandemic: 11 June 2020–11 November 2020;
2. Holiday rise: 11 November 2020–11 January 2021;
3. Holiday drop: 11 January 2021–11 March 2021.

To reduce the influence of other variables, we corrected for the influence of multiple potential confounders. Given the limits imposed by our dataset of 89 counties, we took a hypothesis-driven approach, selecting the three available covariates hypothesized a priori to be most likely to confound the relationship between star ratings and COVID-19 outcomes. We ran panel data regressions with GLS random effects. These use the following form (Equation (1)):

$$y_{it}=\alpha +\overset{k}{\underset{k=1}{\sum }}\beta_{k}x_{ik}+u_{it}$$

where:

- *y_it_ =* COVID-19 outcome accounting for localities, time, covariates, and error;
- *α =* y-intercept;
- *k* = number of covariates;
- *i* = number of localities;
- *t* = observations across time;
- *β_k_ =* coefficient for each covariate;
- *x_ik_ =* time-invariant covariates across localities;
- *u_it_ =* random error varying across localities and time.

We ran the panel data regressions for each of the three COVID-19 outcomes, including the following time-invariant covariates:

- Population infection rate → star rating, age ≥ 65, poverty;
- Population death rate → star rating, age ≥ 65, poverty, no health insurance;
- Infected death rate → star rating, age ≥ 65, poverty, no health insurance.

We considered age ≥ 65 to be relevant for all outcomes, as age appears to be a risk factor for both infection and prognosis [19]. Poverty was considered to be relevant for all outcomes. Taking poverty into account can yield insights into socioeconomic variances and their effects, such as income-related facility resources, rates of working from home, and household size. This, in turn, can impact infection rates, stress-induced immunosuppression (affecting both infection and death), and healthcare access, which impacts death rates [20,21]. A lack of health insurance was considered to be relevant only to deaths, as it may reflect healthcare access and quality of care [22]. Although other factors may significantly affect COVID-19 outcomes, the size of the dataset limited our ability to correct for additional factors. This is addressed further in the limitations section.

Our complete dataset, including localities, daily COVID-19 outcomes, and demographics, contained approximately 100,000 observations, in addition to the 46,000 PORs represented by the 89 LMPORs. We performed the regression analysis in Stata v16, producing the coefficient for each covariate, their respective *p*-values, and confidence intervals.

From the above covariates model, we also modeled a counterfactual comparison group in which the average star rating was increased to 4.0 stars (a 0.3-star increase). This increase was within the bounds of the data available in our model. We estimated the absolute risk reduction (ARR) and relative risk reduction (RRR) for each study outcome by comparing the real-world outcomes seen with the real-world mean star rating to the estimated outcomes predicted by our counterfactual model. 

## 3. Results

### 3.1. Descriptive Data

The 89 localities included in our study provided a significant range of demographics. Comparing the highest and lowest variables on a county-by-county basis, poverty varied nearly five-fold, the rate of uninsurance varied nearly eight-fold, and the rate of those aged at least 65 years varied nearly three-fold (see Table 1). The area with the highest star rating was nearly 1-star higher than the worst-rated area.

Our complete model of the 89 counties captured approximately one-third of U.S. total COVID-19 outcomes and total U.S. population (see Table 2). It provided an accurate representation of U.S. COVID-19 outcomes as a whole, with the observed population infection rate in the 89 localities staying within 6% of the national average across the study period.

### 3.2. Main Results

We found doctor star ratings significantly associated with COVID-19 infection outcomes during the first entire year of the pandemic. We estimated the ARR and RRR for each study outcome by comparing the real-world observations to the estimated outcomes predicted by our model with a 0.3 unit higher average star rating. The increase represents an average rating of 4.0 stars instead of 3.7–56% of healthcare practices measured individually already meet or exceed this goal [23,24]. As shown in Table 3, we found a 16.8% RRR of the infected death rate and a 10.7% RRR of the population infection rate during the first year of the pandemic with a 0.3 increase in LMPOR. Generally, we found a higher likelihood of statistical significance when the time window for analysis was longer, whereas shorter time windows rarely showed a significant association for any COVID-19 outcome, likely due to the decrease in dataset size.

We used the modeled RRRs to estimate the number of COVID-19 outcomes that could have been prevented. Our model predicted that a 0.3 unit higher star rating could have resulted in 87,782 fewer COVID-19 deaths and 3,083,209 fewer infections during the first entire year of the pandemic in the U.S. (see Table 4).

## 4. Discussion

We found a significant association between LMPORs and COVID-19 outcomes. The geographic areas with the most satisfied patients, on average, fared significantly better against COVID-19 compared with areas with the least satisfied patients. Our modeling of a 0.3 unit higher U.S. average star rating predicted 87,782 fewer deaths during the first year of the COVID-19 pandemic, representing a 16.8% higher survival rate for the infected U.S. population, assuming an equal number of infections. During the later pandemic (11 June 2020–11 March 2021), this value was an even higher 20.9%. Although we acknowledge the potential for residual confounding in our model, this higher survival rate might possibly illustrate the ability of the highest rated medical practices to rapidly adapt and respond to a novel infectious disease. Another possibility is that the higher ratings of medical practices may indicate closer physician–patient relationships and greater patient trust in their respective physicians, leading to improved patient behavior or willingness to accept physician recommendations [25], which could include COVID-19-related risk factors, such as weight management. However, a fully nuanced explanation is probably multifactorial. For example, obese patients report greater satisfaction with their healthcare providers than their normal-weight counterparts [26].

We also found LMPORs to be associated with the population infection rate. Our modeling of a 0.3 unit higher U.S. average star rating predicted 3,083,209 fewer infections, representing a 10.7% RRR for the first entire year of the pandemic (and 10.3% RRR for the later pandemic). We did not anticipate this association, since patient satisfaction is not a direct component of the virus’s mechanism of transmission. However, successful preventive healthcare may reduce an individual’s vulnerability to infection and associated adverse outcomes. Furthermore, as trust increases between patient and provider, improved patient behavior is also anticipated [25]. This may indicate a patient’s willingness to adhere to doctor recommendations, such as hand-washing and social-distancing, which in turn reduce infection. We therefore anticipate the association between infection rates and PORs to represent less tangible measures of quality of care, such as patient trust and preventive care. 

Generally, we found a higher likelihood of statistical significance when the time window for analysis was longer, whereas shorter time windows rarely showed a significant association for any COVID-19 outcome, likely due to the decrease in dataset size. We also note that the changing progression of the pandemic and seasonal cultural traditions may have played a role in reducing the statistical significance of the star rating association during the shorter time windows.

The least predictable of the outcomes was population death rate, which was not statistically significant during any of the time windows we measured. However, population death rate is a metric that incorporates into its denominator a significant portion of the population who were not infected. With this in mind, population death rate analysis would not have as much statistical power as the infected death rate analysis, which has only the infected in its denominator. The subsequent analysis of the data extending beyond the first year of the pandemic may provide additional nuance.

### 4.1. Implications

The available evidence shows that patient experience has a positive association with the processes of care for both prevention and disease management [27]. In addition to improved patient behavior, patient experience has also been associated with improved clinical outcomes. For example, the analysis of aggregate data has shown that patient-centered care is associated with lower mortality and lower readmission rates for myocardial infarctions [25]. Similarly, high Yelp ratings are associated to an improvement in clinical outcomes for myocardial infarction and pneumonia [9]. We find that LMPORs serve as a significant predictor of both COVID-19 infection and death. This finding is in agreement with and furthers the available evidence that improvements in patient satisfaction ratings are associated with improvements in clinical outcomes.

It is especially important that our findings not be misconstrued as expressions of contempt towards doctors or other health professionals. Our findings do not fault healthcare providers for adverse outcomes. Rather, our research uncovers additional benefits afforded through the successful pursuit of superior patient experience in healthcare, beyond the direct interactions between doctor and patient. For example, the existing research calls for “active listening” by all members of healthcare practices, including administrative staff [28,29], which may result in increased patient satisfaction, improved patient behavior [25], improved outcomes [28], and earned patient trust [30,31]. Those patient experience improvements are likely to be reflected in star ratings that are here shown to predict health outcomes. Evidence has shown the areas that contribute most to doctors’ happiness seem to focus on the satisfaction of their patients [32], and it is likely that happier doctors lead to improved patient experiences. Americans generally view medical professionals favorably [33] and 78% of patient complaints are not about physicians; therefore, programs that aim to improve patient care and reduce patient dissatisfaction should be directed at the entire staff, not only physicians [29]. The evidence, taken together with our findings here, shows that practices who cultivate a team of caring experts who deliver high patient satisfaction and corresponding star ratings may yield a more unified and satisfied team, an enjoyable working environment, and improved patient outcomes.

Prior to the development of a vaccine, a significant portion of public health policy response to the pandemic was directed towards non-pharmaceutical interventions, including school closures, banning of mass gatherings, isolation of ill persons, disinfection and/or hygiene measures [34], and the mandatory wearing of masks [35]. Although such measures may have reduced infections, “reactive” measures may introduce the risk of other adverse outcomes, such as increased suicidality [36], closure of health practices [37], reduced cancer screenings [38], and are unlikely to have the benefit of “proactive” measures [34]. For example, at the onset of the COVID-19 pandemic in March 2020, appointments for breast, cervical, and colon cancer screenings decreased by 86% to 94% percent compared with average volumes in previous years and comparable times [38]. Some of these systemic issues may be difficult to change. However, our work reinforces that public policy and important efforts within individual practices that facilitate improved patient experience may result in improved patient satisfaction ratings and improved outcomes without those risks to patient or practice.

### 4.2. Limitations

Online reviews are not verified and have an inherent selection bias in the reporting of patient experiences. However, our study only assessed these reviews in aggregate. The available evidence suggests online reviews are in agreement between the various platforms [39], contain important information that can generate insights into quality of care [23], observe aspects of care related to important patient outcomes [9], and mirror many aspects of more traditional surveys, such as the HCAHPS [5].

LMPORs from the HPI dataset may have changed since their original publication in 2014. There has been a general paucity of studies that examine LMPORs, and we found no equivalent and no more recent source from which to obtain LMPORs. Despite the rise of telehealth during the COVID-19 pandemic, patient satisfaction with video visits was high [40]. Further research may obtain a more fine-scale LMPOR association, such as practice-level associations within a hospital, or broad-scale LMPOR associations, such as country-level associations.

The available LMPORs were for the most populous localities within the U.S. The outcomes for those areas differed modestly and showed elevated infection rates, yet better survival rates, than the remainder of the U.S. Therefore, our findings are most applicable to urban areas of the U.S.

Patients likely do not select healthcare providers according to county boundaries. Their selection behavior may be smaller or larger than county borders. The star ratings selection from a 50-mile radius may include portions of multiple counties, or less than an entire county. Some level of border leakiness is inherent in a city- or county-level analysis approach—there will inevitably be patients crossing from one region to another for some of their medical treatment, adding error to the estimated characteristics of patients receiving medical service in a given region.

Our findings include the results of a panel data regression and describe a linear relationship between LMPORs and COVID-19 outcomes. The nature of this form of analysis is sensitive to outliers and may overfit the data. Other statistical approaches in a larger or more detailed dataset may reveal more nuanced results. Furthermore, our analysis was not designed to predict outcomes for patient ratings beyond the extremes of our model, which extend from 3.2 to 4.2 stars.

Our findings are a result of broad trends and may not prove accurate in every individual circumstance and locality. Our findings included anomalous localities that experienced low COVID-19 death rates despite low patient satisfaction rates. 

Although other factors contribute to COVID-19 survivability, our study was limited to a focus on LMPORs. Although a panel of 89 counties vs. all the dates of interest result in a large panel dataset, the correlated nature of day-to-day COVID-19 rates means the dataset was unable to support correction for more than three confounders. We were therefore unable to include all possible confounders, such as certain demographics or governmental or institutional interventions, including gender, race, obesity, population density, or mandates. There also may be some features of healthcare facilities that the patient experience may be blind to, which nonetheless affect COVID-19 outcomes. Nevertheless, given that the size of our dataset only supported the correction of three confounders, we selected the factors deemed to be of highest relevance and objectivity, and with the lowest correlation between each other. For example, although obesity is a significant risk factor for COVID-19 [41,42], it also has a significant overlap with poverty; the highest rates of obesity occur among population groups with the highest poverty rates [42]. A larger dataset would facilitate correction for additional confounders. For example, socioeconomic status (SES) includes poverty and lack of health insurance, for which we were able to correct. However, SES is notoriously difficult to capture, and a larger dataset would allow correction for the larger range of factors that make up SES.

Our analysis was based on aggregate patient data and did not assess individual-level patient data. Individual-level data offers advantages; however, aggregate patient data continues to be the mainstay of systematic reviews and can support clinical practice guidelines [43].

Although PORs may be influenced mostly by the patient–clinician relationship [44], PORs do not directly measure physician clinical skill, and in some cases may be counter to clinical skill. For example, a study found that, although outpatient respiratory tract infections (RTIs) are mostly viral in nature and rarely warrant treatment with antibiotics, patients who received antibiotic prescriptions for respiratory tract infections reported a nominal increase in satisfaction [45]. Furthermore, the totality of patient experience encompasses far more than provider skill. For example, a patient who is unable to book an appointment due to a malfunctioning telephone system may report a lower satisfaction.

A prior analysis of 1.5 million online reviews showed that health practices tend to receive about one-fifth of the quantity of reviews of restaurants and hotels [46], giving each POR more influence on a practice’s overall rating online. In comparison to restaurants, doctors are 64% more likely to receive a 5-star review, but 194% as likely to receive a 1-star review [46]. This suggests negative reviews to be especially important quality indicators for health practices. Rather than viewing negative PORs as misguided criticism, our findings suggest healthcare providers welcome the valuable assessment of the total patient experience. In managing online reviews, practices should avoid self-dealing, review incentives, and other review manipulations, which may be illegal [47] or generate negative publicity [48]. Any individual POR may be inaccurate or false; however, the evidence suggests that, on the whole, PORs do truly reflect patient experiences and outcomes.

The correlations we found do not imply causation; the act of giving a positive review does not itself inoculate against adverse outcomes, and the act of giving a negative review does not itself induce adverse outcomes.

## 5. Conclusions

Our new findings uncover a significant relationship between COVID-19 outcomes and reported patient satisfaction levels. Specifically, the geographic areas with higher patient satisfaction online review ratings benefitted from substantially better COVID-19 outcomes. Prior research has shown that positive patient experiences predict improved myocardial infarction and pneumonia outcomes, among other improvements; these new findings suggest patient online reviews may predict COVID-19 outcomes as well, providing the first illustration of this phenomenon in a pandemic context.

## Figures and Tables

**Table 1:** Basic demographics of the modeled localities.

	Min.	Median	Average	Max.	Standard Deviation
**County Population**	226,941	836,062	1,229,100	10,081,570	1,343,643
**Poverty**	5.60%	14.70%	14.54%	27.50%	4.29%
**Age ≥ 65**	9.20%	13.40%	13.63%	24.30%	2.36%
**No Health Insurance**	3.20%	8.50%	9.23%	27.70%	4.38%
**Stars**	3.20	3.70	3.70	4.15	0.21

**Table 2:** Summary outcomes for the modeled dates.

	11 March 2020	11 June 2020	11 November 2020	11 January 2021	11 March 2021
**Modeled Number of Days**	1	93	246	307	366
**Modeled Number of Counties**	89	89	89	89	89
**Modeled Population**	109,389,862	109,389,862	109,389,862	109,389,862	109,389,862
**US Population**	328,239,523	328,239,523	328,239,523	328,239,523	328,239,523
**Model Pop./US Pop.**	33.326%	33.326%	33.326%	33.326%	33.326%
**Model Deaths Total**	32	35,961	77,656	114,369	169,656
**US Deaths Total**	40	113,073	238,816	369,388	523,420
**Model Deaths/US Deaths**	80.0%	31.8%	32.5%	31.0%	32.4%
**Model Infections Total**	623	698,299	3,609,295	7,868,688	10,128,763
**US Infections Total**	1339	2,010,456	10,286,991	22,265,944	28,731,120
**Model Infections/US Infections**	46.5%	34.7%	35.1%	35.3%	35.3%
**Model Deaths/Model Population**	0.0%	0.0%	0.1%	0.1%	0.2%
**US Deaths/US Population**	0.0%	0.0%	0.1%	0.1%	0.2%
**Model Rate/US Rate**	240.1%	95.4%	97.6%	92.9%	97.3%
**Model Infections/Model Population**	0.0%	0.6%	3.3%	7.2%	9.3%
**US Infections/US Population**	0.0%	0.6%	3.1%	6.8%	8.8%
**Model Rate/US Rate**	139.6%	104.2%	105.3%	106.0%	105.8%
**Model Deaths/Model Infected**	5.1%	5.1%	2.2%	1.5%	1.7%
**US Deaths/US Infected**	3.0%	5.6%	2.3%	1.7%	1.8%
**Model Rate/US Rate**	171.9%	91.6%	92.7%	87.6%	91.9%

**Table 3:** Star-rating association to COVID-19 outcomes *.

	Model Avg. Incidence **				+0.3★ ARR			+0.3★ RRR		
	Actual	+0.3	95% CI		Est.	95% CI		Est.	95% CI	
**Pandemic Year (11 March 2020–11 March 2021)**										
**Infections/Population**	3.04%	**2.72%**	**2.46%**	**2.97%**	**0.33%**	**0.58%**	**0.07%**	**10.73%**	**19.17%**	**2.29%**
**Deaths/Population**	0.06%	0.04%	0.01%	0.08%	0.01%	0.05%	−0.02%	25.14%	84.07%	−33.79%
**Deaths/Infections**	2.61%	**2.17%**	**1.93%**	**2.40%**	**0.44%**	**0.67%**	**0.20%**	**16.79%**	**25.78%**	**7.79%**
**Early Pandemic (3 November 2020–6 November 2020)**										
**Infections/Population**	0.29%	0.20%	0.05%	0.36%	0.08%	0.23%	−0.07%	28.56%	81.16%	−24.04%
**Deaths/Population**	0.01%	0.01%	−0.02%	0.04%	0.01%	0.04%	−0.03%	40.58%	263.76%	−182.61%
**Deaths/Infections**	3.46%	3.16%	2.62%	3.71%	0.30%	0.84%	−0.25%	8.56%	24.32%	−7.20%
**Later Pandemic (6 November 2020–3 November 2021)**										
**Infections/Population**	3.99%	**3.58%**	**3.24%**	**3.91%**	**0.41%**	**0.75%**	**0.07%**	**10.32%**	**18.81%**	**1.83%**
**Deaths/Population**	0.07%	0.06%	0.01%	0.10%	0.02%	0.06%	−0.03%	24.07%	85.00%	−36.85%
**Deaths/Infections**	2.32%	**1.84%**	**1.58%**	**2.09%**	**0.48%**	**0.74%**	**0.23%**	**20.89%**	**31.80%**	**9.98%**
**Summer/Fall Pandemic (6 November 2020–11 November 2020)**										
**Infections/Population**	1.90%	1.69%	1.37%	2.00%	0.21%	0.52%	−0.10%	11.06%	27.62%	−5.50%
**Deaths/Population**	0.05%	0.04%	−0.01%	0.09%	0.02%	0.06%	−0.03%	29.24%	125.76%	−67.28%
**Deaths/Infections**	2.84%	**2.19%**	**1.82%**	**2.56%**	**0.65%**	**1.02%**	**0.28%**	**22.99%**	**36.03%**	**9.95%**
**Holiday Rise (11 November 2020–1 November 2021)**										
**Infections/Population**	5.06%	4.69%	3.88%	5.49%	0.37%	1.17%	−0.43%	7.31%	23.19%	−8.57%
**Deaths/Population**	0.08%	0.06%	−0.04%	0.16%	0.02%	0.12%	−0.08%	23.05%	145.02%	−98.91%
**Deaths/Infections**	1.73%	1.37%	0.91%	1.83%	0.37%	0.83%	−0.10%	21.10%	47.71%	−5.50%
**Holiday Drop (1 November 2021–3 November 2021)**										
**Infections/Population**	8.26%	7.29%	6.29%	8.28%	0.97%	1.97%	−0.03%	11.76%	23.86%	−0.33%
**Deaths/Population**	0.13%	0.10%	−0.02%	0.23%	0.02%	0.15%	−0.10%	19.32%	119.12%	−80.47%
**Deaths/Infections**	1.56%	1.38%	0.93%	1.83%	0.18%	0.63%	−0.27%	11.27%	40.08%	−17.53%

* Bold values are statistically significant at *p* < 0.05. ** Average incidence values across panel data regressions render an average point estimate across the time window, which is not equivalent to an incidence calculation performed cross-sectionally on data from the last day of the series. ★ represents POR star ratings for the model.

**Table 4:** Year-one COVID-19 pandemic outcomes with a 0.3 star improvement *.

		+0.3 ★Modeled Outcomes			Difference (Actual–Modeled)		
Pandemic Year (11 March 2020–11 March 2021)	Actual	Estimate	95% CI		Estimate	95% CI	
Infections of US Population	28,729,781	**25,646,572**	**23,221,551**	**28,071,593**	**3,083,209**	**658,188**	**5,508,230**
Deaths of US Population	523,380	391,807	83,364	700,249	131,573	−176,869	440,016
Deaths of US Infected	523,380	**435,518**	**388,440**	**482,596**	**87,862**	**40,784**	**134,940**

* Bold values are statistically significant at *p* < 0.05. ★ represents POR star ratings for the model.

## Data Availability

All data generated or analyzed during this study are included in this published article. Supplementary datasets used and/or analyzed during the current study are available from the corresponding author upon reasonable request.

## Supplementary Materials

The following supporting information can be downloaded at: https://www.mdpi.com/article/10.3390/healthcare11101411/s1.
